# The Heavy Metals in Agrosystems and Impact on Health and Quality of Life

**DOI:** 10.3889/oamjms.2015.048

**Published:** 2015-06-03

**Authors:** Adnan Tutic, Srecko Novakovic, Mitar Lutovac, Rade Biocanin, Sonja Ketin, Nusret Omerovic

**Affiliations:** 1 *International University of Travnik, Travnik, Bosnia and Herzegovina*; 2 *Solid, Subotica, Serbia*; 3 *University of Union, Serbia*; 4 *State University of Novi Pazar, Vuka Karadzica bb, Novi Pazar, Serbia*; 5 *University of Novi Sad, Trg Dositeja Obradovica 6, Novi Sad, Serbia*; 6 *International University of Brcko, District Brcko, Bosnia and Herzegovina*

**Keywords:** life environment, agroecosysems, heavy metals, mercury, toxicity, metabolism, chain pollution, chemical protection

## Abstract

The metal is a chemical element that conducts electricity well and heat, and the nonferrous metals builds cations and ionic bonds. Heavy metals include metals whose density is higher than 5 g/cm^3^. The whole range of the metal is in the form of essential trace elements, essential for a number of functions in the human body, and its deficiency results in a lack of occurrence of a serious symptom. The best examples are anemia lack of iron, lack of chromium in diabetes, growth problems in lack of nickel. Other elements such as lead, cadmium, mercury, arsenic and molybdenum have been shown to exhibit large quantities of toxic effects. The paper examines the problem of heavy metals originating from agriculture on agroecosystems. This group of pollutants is considered the most important cause of degradation of soil quality, surface and groundwater and direct causal adverse effects on human and animal health. In order to complete the environmental monitoring of pollutants, these main categories, origins, and possible negative impacts of the basic principles of preventing their toxic effects were examined.

## Introduction

In chemistry, a metal (from the Greek “μέταλλον” - métallon, mine) is a chemical element, compound or alloy that has a characteristic of high electrical conductivity. In metals, the atoms release electrons and build positive ions (cations). These ions are surrounded by delocated electrons, which are responsible for the property of conductivity. The solid body that is built in this way is held by electrostatic interactions between ions and the electron cloud, and such a bond between atoms is called the metallic bond.

Metals are sometimes described as a set of positive ions surrounded by sea of the delocated electrons. They are one of three groups of elements if viewed by the characteristics of ionization and atomic bonds, in addition metalloids and non-metals. The term heavy metals include metals whose density is greater than 5 g/cm^3^. The whole set of these metals in the form of trace elements are necessary, essential for numerous functions in the human body, and their lack leads to the occurrence of severe deficiency of the symptoms. The best examples are anemia in the absence of iron, diabetes in the absence of chromium, growing problems in the absence of nickel. In some elements, such as arsenic and nickel, the function is not yet fully researched. In other elements such as lead, cadmium, mercury, arsenic and molybdenum have been proven that in large quantities they indicate toxic effect.

### The agroecosystems and the negative impacts

Agroecosystems are pieces of land where man cultivates different kinds of cultural plants used in food or in industry. In addition to cultivated plants, in these ecosystems still have been living and different types of wild plants, animals, fungi and micro-organisms.

Agroecosystems are formed in places of natural ecosystems. The land is cultivated, and on it are sown or planted cultures of different plants. Depending on what kind of cultures is cultivated we distinguish fields, vineyards and orchards. In the fields are cultivated herbaceous crops: corn, sunflower, beans, potatoes, tomatoes, peppers, tobacco, wheat, rye, rice, oats, peas, soybeans, lentils, cabbage. In the vineyards are grown different varieties of grape vines, and in orchards woody cultures.

While in natural terrestrial ecosystems climate plays a decisive role in the formation of the living communities, but in agroecosystems man is the most important environmental factor. He chooses which plant will be the main producer of food in agrosystem. He irrigates or dries out the land in accordance with the needs of the plant that is grown. His insufficiently fertile land cultivates and enriches. The susceptible plants to frost and cold he protects (in the greenhouse).

Huge efforts and costs that man makes in order to eliminate from his fields all the other plants and animals and forms a completely pure monoculture (fields where it is grown only one vegetable crops), almost there’re no agro-ecosystem in which, in addition to crop plants, there’re no “undesirable” supporting residents. However, one part of these “undesirable” residents man has a great benefit [1, 2].

The „hazardeous” members of the agrobiocenosis are: weeds, parasites, herbivores, insects, birds and rodents [3]:

1. weedy plants make damage taking a part of space, water, minerals and the light of cultural plants, which inevitably reduce the overall production. If a man does not fight against them they can completely displace cultural plants from agroecosystem. In our fields most common weedy plants are: cockle, ponceau, bonito, cinderella, pigweed, curly dock, pea and burdock;
2. parasites are organisms that live at the expense of others. These are the most common fungi, bacteria and viruses. They cause a lag in growth and development, reducing the yield to extinction. The most common parasites in our cultures are: cereal rust on wheat, garki on corn and mildew on grapevines or apple;
3. insects feed on different parts of crops and produce great harm. Harmful insects in our fields are: potato beetle, cereal ground beetle, moth, roach, plant lice, larvae chafer;
4. rodents are a nuisance to the wheat fields. These are: mice, voles, rabbits, hamsters;
5. birds that live in flocks and feed on plant foods can sometimes produce damage in agroecosystems. In our conditions are the most numerous: starlings, garki, partridges and quails. However, birds also destroy other pests (mainly insects and rodents), and the damage of them is much smaller than benefit - starlings, for example, also feed with roaches.

„Useful” members of agrobiocenosis – in agrobiocenosis live or regularly visit and some animals from which a man has big benefits:

1. earthworms and other soil organisms are very useful members of the agrosystem. Digging channels and feeding with detritus earthworms enrich and ventílate land, which has a positive effect on plants that are grown;
2. insect pollinators such as bees, bumblebees and butterflies can not imagine any biocenosis, because without them there would be no useful fruits;
3. insects carnivores and songbirds such as wasps, hornets, ladybug, titmice and their larvae eat in one year a huge amount of harmful insects, without them the yield on the fields would be extremely lower;
4. owls, birds of prey and foxes are the most useful members of the agrobiocenosis, because on average eat the most harmful members of the agrobiocenosis.

Agroecosystem as the belonging factor of the biosphere and anthrophosphere represents not only an important source of energy and substance, rather than the primary recipient of numerous residual waste pollutants [4]. From the hygienic quality of soil depends on the sustainability of the habitats of flora and fauna, the quality of groundwater and surface water, the welfare of domestic animals, as well as the health of human population. Substances that can disrupt the natural ecosystems of land, water and air are called hazardous substances. The most common hazardous toxicants of the agroecosystems are heavy metals, radionuclides, synthetic organic substances and pesticides [5-9, 15-18]. Due to increased concentrations of toxicants in soil and water it leads to their accumulation in plants, and thus in the food chain of animals and man. Some of them are of little toxicological significance, while others are highly toxic and produce a range of health problems. Among the most harmful nutrients for human health, as the end consumers of plant and animal products include the heavy metals.

Heavy metals in agroecosystems runs a series of chain reactions that cause a change in the quality of soil, water and the atmosphere, which is inevitably reflected in the changes in the structure of living organisms that inhabit them. In addition of ending up of the heavy metals in soil of the parent substrate during pedogenesis and industrial plants pollute the air with heavy metals, and therefore pollution is transferred to land and water [11-14]. In the surface horizons of agricultural land can often be found and heavy metals that are not already geochemical but of anthropogenic origin as a result of various human activities. Prolonged intake of pollutants in the soil can lead to a reduction of its buffering capacity as such can cause permanent contamination of soil and groundwater.

### Sources, origin and toxicity of heavy metals

The most common question of toxicity actually is a question of quantity, but this range varies greatly with each element. Thus, the daily necessary amounts of cobalt, the central atom of vitamin B12, which is necessary in the formation of erythrocytes, is moving about 0.1 microgram. In an amount of 25-30 mg/day occur the symptoms of poisoning comprising difficulties by the gastrointestinal tract, heart, and kidney damage. Other elements, for example such as thallium, are toxic in any amount. The table lists certain branches of industries that emit heavy metals.

**Table 1:** Industrial branches – emitters of heavy metals.

Industrial branch	Cd	Cr	Cu	Hg	Pb	Ni	Sn	Zn
Paper industry	-	+	+	+	+	+	-	-
Petrochemistry	+	+	-	+	+	-	+	+
Production of clorine	+	+	-	+	+	-	+	+
Fertilizer industry	+	+	+	+	+	+	-	+
Ironworks and steelworks	+	+	+	+	+	+	+	+

Heavy metals may be in the form of fine dust particles to end up in the atmosphere, where they are precipitated in water and soil. In the water they’re quickly diluted and precipitated as sparingly soluble carbonates, sulfates, sulfides, or at the bottom of the water surface. When the adsorption capacity of sediments is exhausted, the concentration of metal ions is increased in the water. Circulation of heavy metals in nature is highly dependent on changes which are subject to these metals. Toxicity of heavy metal in particular increases in the chelating process and creation of sulfide with biologically active substances, particularly enzymes. This procedure is called biomethylation. A special toxicity exhibit organometallic compounds of mercury, lead, chromium and selenium.

Increased concentrations of heavy metals can be a cause of occurrence of the autoimmune diseases, while creating proto bodies directed against their own organs. The most common examples are the different types of allergies, and women interference with the function of corpus luteum of the ovary, which prepares the mucous membrane of the uterus for implantation of a fertilized egg.

It is assumed that the heavy metals will also affect the metabolism of zinc, thereby provoking its lack. Zinc deficiency can disrupt the function of the pituitary, thyroid, adrenal glands, ovaries and testes, which may adversely affect fertility.

From the stories of heavy metals is quite clear that we are somehow permanently and fatefully exposed to various environmental factors pollution. Of course that a worker in the chemical industry can choose another job, as we can change furniture or remove plywood from flat.

Despite all this, we still will be exposed to harmful substances from the carpet or carpet impregnated because of moths or from our clothing made of cotton treated with pesticides. The list is broader and if we read materials that are part of cosmetics and adhesives.

When these substances once get into our bodies, they’re accumulated in the fatty tissue, as well as in the liver, kidneys and brain, and from there demonstrate the operation of the biochemical and hormonal processes, such as metabolism and cell growth, and fertility. Heavy metals are different from other contaminants in that that people neither produce nor can destroy them and they are not created by micro-organisms.

Sources of heavy metals are:

1. waste products of numerous industrial processes (cellulose industry releases mercury);
2. urban settlements (sewerage water; lead on roads);
3. heavy metals are integral part of various pesticides and anti-corrosive paint.

Primary contamination is: contamination of plants through soil, wáter, air, fertilizer, contamination of animals with food or water.

Secondary contamination is: during processing, packaging, storage, through devices and packaging, cutlery, pots, additives

Effects on organisms:

1. Zink and copper denatures proteins;
2. Copper blocks blood respiratory pigments;
3. Mercury and lead effect on nervous system;
4. Cadmium effects on the function of the kidney.

Organisms have the ability to form organic complexes with metals and in this way make them less toxic (exception is mercury, which is just the most toxic in the organic complex - methylmercury). The most important (classic) inorganic contaminants are As, Hg, Pb, Cd.

Mobility of heavy metals from anthropogenic pollution depends on the acid reaction, content of organic matter and humus, physical granulation, temperature and soil moisture. The most common heavy metals in soil and water are arsenic (As), cadmium (Cd), chromium (Cr), mercury (Hg), zinc (Zn), nickel (Ni), lead (Pb) and vanadium (V). Heavy metals in agroecosystems originate from fertilization with organic and mineral phosphorus fertilizers, from industrial plants, mines, power plants, pesticides, urban waste water and exhaust fumes of cars.

Determination of the concentration of input and output influx of heavy metals in agriculture, knowing the possibilities of their degradation or inactivation is an essential precondition for sustainable management of these toxicants in agricultural systems. Agrosystem can be seen as a transitional form between natural and standard ecosystems that are under the direct influence of human activities.

Statutory legislation and international legal framework of many countries clearly define the maximum concentrations of heavy metals in the agrosystem. Restrictions apply to all media of agricultural products in order to reduce the potential risk to phytotoxicity plants and human health as the ultimate consumers.

The adoption of legal measures contributed to the partial reduction of industrial emissions, however, the overall rate of intake of most metals in soil and aquatic systems is not reduced, but even increased. Enormously high concentrations of certain heavy metals are characteristic of urban and developed industrial areas (Moolenaar, 1998; Guinee et al. 1999) [15, 16]. According to the EU Directive on environmental protection and land (86/278/EEC) Member States [17] must comply with the established limit values for heavy metals in soil, which are shown in Table 2.

**Table 2:** Limit values of heavy metals (mg/kg).

Parameters	Limit values
Cadmium	1-3
Copper	50-140
Nickel	30-75
Lead	50-300
Zink	150-300
Mercury	1-1,5

Member States may allow exceeding the threshold values of these parameters on land that has a constant pH value greater than 7. The maximum allowable concentrations of these heavy metals must not exceed this value more than 30%. Member States must also ensure that there is no resulting hazard to human health or the environment, especially groundwater.

*Mercury* is the only liquid metal. Natural source of mercury represents the evaporation of the earth’s crust (the bottom of the ocean, river, and land). It exists in lamentable form as Hg (I) and Hg (II) compounds, in the organic or inorganic form. Methyl-mercury and other organic compounds of mercury are formed as a product of microbiological activity (methane bacteria) in an aqueous environment.

Much has been researched, written and discussed on the toxicity of mercury. In the scientific community took place several so-called amalgam wars in which the amalgam advocates sought to prove its harmlessness and justification of the application, while the opponents were trying to achieve a legal ban on amalgam in dental medicine. Even today, there are very timely discussions on the potential and actual harmful effects of dental amalgam and to the possible limitation of their application. The views are still controversial, and the use of amalgam in dental medicine controversial.

It is certain that elementary mercury has a toxic effect on the human body and its actions can damage the nervous system, kidneys, liver, digestive system and glands with internal secretion.

Toxic effect of mercury is also associated with neurodegenerative diseases, autoimmune disorders, disorders during pregnancy and childbirth, and a range of mental disorders. For toxic effect is sufficient continuous exposure in very low doses. Therefore, it is reasonable to ask a question: is the amount of mercury released from amalgam plants sufficient to achieve some of the toxic effects?

The numerous studies that have been carried out resulted in inconsistent and contradictory results. On the one hand, convincing evidence confirms that the concentration of mercury in the body increased in people who have amalgam fillings (measured by the amount of mercury in the brain, liver, kidney and urine), while, on the other hand, has not been demonstrated a clear causal-consequental link between mercury from dental amalgam and some of the chronic diseases it can cause.

Taking supplements of vitamin E and selenium in combination can reduce the toxicity of mercury found in fish, according to a new study conducted on laboratory rats. Conflicts over the fact whether the health benefits of consuming omega-3 of fatty acids from fish is greater than the dangers of mercury poisoning however, outweigh the benefit of omega-3 of the fatty acids. They are directly associated with improved fetal development, cognitive function and heart health of the unborn child. Methyl-mercury is a contaminant found in different concentrations in all kinds of fish. It became alarmingly dangerous to health, so the FAO/WHO made the decision that the provisional tolerant weekly dose is of 1.6 mg/kg of the body weight. The researchers found that supplements containing vitamin E and selenium in combination reduce mercury toxicity in laboratory rats. These results indicate that the antioxidants in the diet can alter the toxicity of the methyl-mercury.

This study is of great importance because according to the Environmental Protection Agency in the United States, one of six pregnant women have blood mercury concentrations sufficient to cause fetal death, so the effect of supplements could reduce the risk of 650 000 unborn children. Until further notice, the US FDA recommends that pregnant women do not eat more than two servings (340 g) of fatty fish a week. An alternative to fresh fish for pregnant women can be omega-3 supplements, most of which were examined by test of the mercury contamination. All mercury compounds are extremely toxic to plants and animals. Phytotoxicity of mercury does not represent a greater environmental problem. The concentration at which exhibited symptoms of excess mercury in plants is significantly above those in normal conditions found in the soil. In addition, the availability of mercury in the soil for plants is usually low. It is believed that the root represents an obstacle to greater accumulation of mercury in the shoot. According to studies, by Beauford (1970) [18], accumulation of mercury in the bud is twenty times higher than in shoot.

The concentration of mercury in plants ranges on average from 10 to 200 ng/g of dry substances, while living near the site of 500 to 3,500 ng/g. For wheat, the concentration of mercury is from 3 to 10 times lower in grain than in straw. The grains of barley and wheat, mercury concentration are around 1 to 2 ng/g of dry substances. Mercury disrupts the structure of biomembranes and changes the activity of enzymes which impairs metabolism and inhibits the growth and development of plants.

**Table 3:** The presribed values of mercury in the world.

Origin of rules	Media	Value	Size
Canada	Drinking water	1 μg/l	MAC

Canada	Fish	0.5 ppm total mercury	

WHO	All sources	0.47 μg/kg TM daily as methyl-mercury 0.71 μg/kg TM daily methyl-mercury + inorganic	TDI

WHO	Drinking water	0.001 mg/l total	GV

JECFA	All sources	5.0 μg/kg TM weekly as total mercury 3.3 μg/kg TM weekly as methyl-mercury	PTWI

### The heavy metals in agroecosystems as consequence of action of the waste waters

Water pollution is the most complex global environmental problem. Any pollution emitted into the environment reaches the groundwater, rivers, lakes and seas. Soil contamination is flowing to the surface and underground water flows. Rivers and lakes are under constant pressure from wastewater pollution from urban areas, chemical waste from industry and transport, pesticides from agricultural areas, etc.

Drinking water reserves are found in surface and groundwater flows and their pollution leads to a reduction of available drinking water supplies. The problem of wastewater creates the problem of drinking water and therefore affects the health of present and future world population. Population, who drink contaminated water, due to an increase of nitrate in groundwater and surface water, is exposed to the risk of cancerous diseases of the digestive tract. This is just one example of the disastrous consequences of lack of wastewater treatment and their direct discharges into watercourses.

Water pollutants can be:

1. chemical (acids, alkali, various salts, pesticides, detergents, phenols, etc.);
2. biological (bacteria, viruses, algae, barnacles, lignins, etc.); and
3. physical (heat, colour, odour, radioactivity, suspended solid substances, sand, mud, etc.).

The main goal of any treatment of wastewater is it’s as fully as possible release of unwanted components - pollutants, which is achieved by applying one or more basic basic processes. One or more of the basic processes that is used to achieve a certain processing effect, form the processing line. More processing lines form the system processing, respectively the system for water quality treatment.

The systems for treatment of wastewater consist of several processing lines (each line treatment consists of various treatment processes):

There is no uniform system for wastewater treatment (WA) because each WA has special characteristics, which is specifically related to industrial WA.

The following basic procedures are used:

1. previous mechanical treatment,
2. physical-chemical treatment and
3. biological treatment.

With the mechanical (or physical) purification are removed: coarse impurities, inert material and a portion of biodegradable substances from waste water. Mechanical purification is done by means of: grids, screens, precipitator for sand, grease trap primary (previous) precipitators and pools for equalizing the flow and composition of waste water (when these are significantly changed during the day). Primary sedimentation allows the deposition of discrete organic and inorganic suspended particles which are separated from the system in the form of sludge. Biological wastewater treatment involves the removal of dissolved organic substances and colloidal non-deposited particles. In the system of wastewater treatment biological treatment takes place as a secondary treatment, and after mechanical or primary treatment [19]. Biological treatment is applied for:

- Removal of organic matter;
- Removal of nitrogen (as biogenic element) by means of nitrification and denifitrication procedure;
- Degradation of primary sludge from the primary process of waste water treatment;
- Degradation of secondary sludge from the process of biological treatment of waste water by a process of stabilization of sludge or digestion. Biological processes of water purification can be carried out as aerobic and anaerobic, with the help of aerobic or anaerobic microorganisms. The difference is in the paths for the biological oxidation of organic matters.

### Heavy metals as consequence caused by the action of waste

By the incineration of waste is obtained energy and reduced fossil fuel consumption. It is believed that it can get up to 10% of energy from waste from the whole of the required energy of a country [22]. The greatest proportion of waste incineration is in Japan. Energy is most commonly used to heat water and dissipation of hot water or steam to users. Waste incineration, solves one problem but creates others: air pollution, water pollution from cooling ash and generation of solid waste [23]. As pollutant substances during the waste incineration occur: chlorine, fluorine, chromium, nickel, zinc, lead, sulfates, etc., and the type and composition of pollutants depend on the composition of the waste. Pyrolysis of waste is a process that is achieved by the hot gases without oxygen. Then organic matter is subject to pyrolysis giving gaseous products and the charred remains. Gas produced by pyrolysis consists mainly of methane, carbon (II) oxide, carbon (IV) oxide and hydrogen gas. This gas is subject to combustion and used for heating. Thereafter the waste material that can burn will be seperated what can burn and create briquettes that can burn in small boiler rooms, and larger amounts in dedicated furnaces for the production of energy for space heating.

One of the most common pollutants in the working environment is the ash. It comes from coal of power plants when working with hazardous polluting the land, water and air. Industrial waste is any waste material generated in the industrial process. This waste can be: inert and dangerous. Inert industrial waste is fully or after separation of certain components can be disposed of in municipal landfills. Certain industries have various participations in the creation of industrial waste. Industrial waste can be: solid, liquid, sludge and unidentified. In industrial production, energy, agriculture, communal activities, processing of raw materials of natural and synthetic origin and in various other activities occur waste materials that can be: a highly toxic, carcinogenic, corrosive, explosive and irritating, and as such represent hazardous waste. Composition of industrial landfill depends on the very industry. From industrial landfills may appear harmful and hazardous elements for the environment: As, Cr, Sr, Mn, Pb, Ni, Zn.

Many landfills are wild and are not registered, and after their leaving in their place may arise village, orchard, garden, or something else. Industrial waste containing stable materials, such as: plastics, rubber, glass and porcelain debris, rubble and the like, are disposed of in landfills that are at least controlled and

Represent large sanitary and health problems. Uncontrolled by man also affect the agroecosystems.

### Estimation of the concentration of heavy metals

*Discrepancy (unadjusted) factor* - Discrepancy indicator compares the input rate of heavy metal concentrations defined with a total output rate acceptable for that system. It is based on existing qualitative standards for individual crops and groundwater quality. If the sum of the input level of the metal exceeds the sum of all outputs discrepant factor is higher, indicating a potential hazard to the environment. By comparing the disturbed equilibrium of different metals can be determined with certainty that which of the heavy metal can lead to the largest pollution of agroecosystems. Also this factor enables to determine the toxicity of priority between different metals.

F_d_= A/(U_c_+L_c_)

F_d_- discrepancy factor; A- sum of all inputs of heavy metals; U_c_- máximum aceptable level of presentation of crops; L_c_- máximum aceptable level of groundwater runoff.

*The sustainability factor* - In the case of limited data on the mobility of heavy metals in the land and the aquatic environment of agroecosystems to assess their toxicity is used the factor of sustainability. This indicator is based on static and already determined results of toxicity and mobility of metals in laboratory conditions.

F_d_= A/(U_c_+L_c_)

F_c_- factor of sustainability; F_e_ – factor of ecological toxicity; F_u_- adoption of crops; F_s_- swelling of groundwater.

*Sustainability of time* - Despite being described indicators provide insight into the dynamic balance of heavy metals, they do not provide information about the date of occurrence of agroecosystem disruption balance. On the basis of calculating the sustainability factor of time can be obtained timely information about the beginning and the length of the validity period of the toxic effects of heavy metals in soil, water and crops.

T_c_= min(T_e_+T_u_+T_s_)

Tc- factor of time sustainability; Te- the start time distortion of agroecosystem standard; Tu- overrun time of metal content in crops; Ts- time exceeding the content of metals in groundwater.

All sustainability indicators of agroecosystems based on dynamic balance of heavy metals and they allow comparison of different agro-ecosystem without having a detailed introduction to each particular chemical processes.

### Statistical methods

Quantitative statistical analysis was performed on the computer. To enter, ranking, clustering, tabular and graphical presentation of the data was used Excel program from the Microsoft Office 2003 software package. The calculations are performed using the SPSS program version 18.0. In all analyzes the limits of statistical significance as the default estimation error of 0.05 or 5%.

Comparison of mortality rates between municipalities was conducted Chi square test, or Fisher exact probability of the null hypothesis (Fisher exact test) in cases where some of the characteristics of the expected frequency was less than five.

Comparing the values of the regression coefficients, for expressing the trend change in the value of mortality rate over the period of 21 years in the municipalities Pčinj district, committed t test.

## Results

In 1988 the territory Pčinj rate is 217 deaths of infants, infant mortality in the municipality of Bujanovac was 51 ‰ and was significantly higher than in Vladicin Han (18 ‰, p <0.05), Vranje (27 ‰, p <0, 05) and Marketplaces (0 ‰, p <0.01). In this year, the highest rate of infant mortality was recorded in the municipality of Presevo (67 ‰) and it was significantly higher than in Bosilegrad (16 ‰, p <0.05), Vladicin Han (p <0.01), Vranje (p < 0.001), Surdulica (28 ‰, p <0.01) and Marketplaces (0 ‰, p <0.01).

In 1989, the highest rate of infant mortality was recorded in the municipality of Bujanovac, which amounted to 50 ‰ and was significantly higher than in Vladicin Han (18 ‰, p <0.05) and Vranje (27 ‰, p <0.05). In the same year the infant mortality rate in Presevo municipality was 42 ‰ and was significantly higher than in Vranje (p <0.05).

In 1990, the highest rate of infant mortality was recorded in the municipality of Presevo (42 ‰) and it was significantly higher than in Vranje (27 ‰, p <0.05) and Surdulica (13 ‰, p <0.05). This year, the infant mortality rate in the municipality of Bujanovac was 36 ‰ and was significantly higher than in Surdulica (p <0.05).

And in 1991, the highest rate of infant mortality was noted in the Presevo municipality (36 ‰) and it was significantly higher than in Bosilegrad (0 ‰, p <0.05), Vladicin Han (10 ‰, p <0.05) and Vranje (13 ‰, p <0.001). This year, the infant mortality rate in the municipality of Bujanovac was 32 ‰ and it was significantly higher than in Vladicin Han (p <0.05) and Vranje (p <0.01).

In 1992, the highest rate of infant mortality was recorded in the municipality of Bujanovac, where it was 44 ‰ and was significantly higher than in Vladicin Han (12 ‰, p <0.05) and Vranje (17 ‰, p <0.001). In the same year the infant mortality rate in Presevo municipality was 31 ‰ and was significantly higher than in Vranje (p <0.05).

In 1993, the highest rate of infant mortality was recorded in the municipality of Presevo (44 ‰) and it was significantly higher than in Vladicin Han (15 ‰, p <0.01), Vranje (28 ‰, p <0.05) and Surdulica (12 ‰, p <0.01).

In 1994, the highest rate of infant mortality was recorded in the municipality of Bujanovac, which was 34 ‰ and was significantly higher than in Vranje (15 ‰, p <0.01) and Surdulica (6 ‰, p <0.01). In the same year the infant mortality rate in Presevo municipality was 28 ‰ and was significantly higher than in Vranje (p <0.05) and Surdulica (p <0.05).

In 1995, the highest rate of infant mortality was recorded in the municipality of Bujanovac, where it was 39 ‰ and was significantly higher than in Vladicin Han (8 ‰, p <0.05) and Vranje (21 ‰, p <0.05).

In 1996, the highest rate of infant mortality was recorded in the municipality of Bujanovac, where it was 33 ‰ and was significantly higher than in Vladicin Han (0 ‰, p <0.01), Vranje (18 ‰, p <0.05) and Surdulica (8 ‰, p <0.05). In the same year the infant mortality rate in Presevo municipality was 23 ‰ and was significantly higher than in Vladicin Han (p <0.05).

In 1999 there was a large fall in the infant mortality rate in Presevo municipality to 6 ‰, this value is significantly lower than in Bosilegrad (28 ‰, p <0.05), Bujanovac (16 ‰, p <0.05) and Surdulica (29 ‰, p <0.01). In 2007, the highest rate of infant mortality was recorded in the municipality of Vladičin Han, which amounted to 31 ‰ and was significantly higher than in Bujanovac (4 ‰, p <0.05), Vranje (5 ‰, p <0.01) and Presevo (5 ‰, p <0.01).

In other years of the period there were no statistically significant differences in the values of infant mortality rate were compared between municipalities.

**Figure 1:**
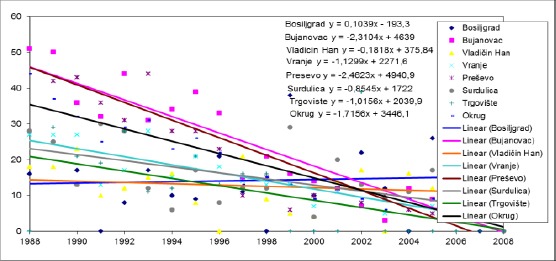
*Trends in infant mortality rate in the municipalities of Pcinja District from 1988 to 2008*.

In the period from 1988 to 2008, there is a trend decline in the value infant mortality rate in all municipalities Pcinj, except in the municipality Bosiljgrad where the average annual rate rose to 0.1039 ‰.

The highest average annual decline in the infant mortality rate of 2.4623 ‰ recorded in the Presevo municipality and this trend was not significantly different from the trend in the municipalities Bosiljgrad (p <0.001), Hani Han, where the average annual decline rate was 0.1818 ‰ (p <0.001), Vranje, where the average annual decline rate was 1.1299 ‰ (p <0.001), Pirot, where the average annual decline rate was 0.8545 ‰ (p <0.001) and Targovishte, where the average annual decline rate was 1.0156 ‰ (p <0.01).

The average annual decline in the infant mortality rate in the municipality of Bujanovac was 2.3104 ‰, and this trend was not significantly different from the trend in the municipalities Bosiljgrad (p <0.001), Hani Han (p <0.001), Vranje (p <0.001), Surdulica (p <0.001) and Targovishte (p <0.01).

The change in Bosilegrad was significantly different from the trend in the municipalities of Vranje (p <0.01), Pirot (p <0.05) and Targovishte (p <0.05).

The change in Vranje was significantly different from the trend in Vladicin Han (p <0.01).

The changes were compared between other municipalities have not happened in significantly different ways.

In 1988 the infant mortality rate 0-6 days of age in Presevo municipality was 18 ‰ and was significantly higher than in Bujanovac (8 ‰, p <0.05).

Already in 1989, the mortality rate of infants aged 0-6 days in Presevo municipality has dropped to 6 ‰ and was significantly lower than in Surdulica (19 ‰, p <0.05) and the market (29 ‰, p <0.05).

In 1990 the highest mortality rate of infants aged 0-6 days and recorded in the municipality of Vranje (16 ‰) and it was significantly higher than in Bujanovac (3 ‰, p <0.01), Vladicin Han (0 ‰, p < 0.05) and Presevo (5 ‰, p <0.01).

In 1991, the infant mortality rate 0-6 days of age in the municipality of Surdulica (20 ‰) was significantly higher than in Bujanovac (4 ‰, p <0.05).

In 1994, the mortality rate of infants aged 0-6 days in the municipality of Hani Han (16 ‰) was significantly higher than in Bujanovac (3 ‰, p <0.05).

In 1997, the lowest mortality rate of infants aged 0-6 days recorded in the municipality of Presevo (0 ‰) and was significantly lower than in Bosilegrad (7 ‰, p <0.01), Vladicin Han (8 ‰, p <0, 05) and Vranje (8 ‰, p <0.01).

In 1998, the mortality rate of infants aged 0-6 days in Presevo municipality was 4 ‰ and was significantly lower than in Vranje (11 ‰, p <0.05) and Surdulica (8 ‰, p <0.05). In 1999, the infant mortality rate 0-6 days of age in the municipality of Presevo (1 ‰) was significantly lower than in Vranje (8 ‰, p <0.05) and Surdulica (21 ‰, p <0.05).

In 2000, the mortality rate of infants aged 0-6 days in Presevo municipality (0 ‰) was significantly lower than in Bujanovac (6 ‰, p <0.05).

In 2001, the infant mortality rate 0-6 days of age in the municipalities of Bujanovac and Presevo (0 ‰) were significantly lower than in Vranje (5 ‰, p <0.05) and Surdulica (12 ‰, p <0.05).

In 2002 and 2004, the mortality rate of infants aged 0-6 days in Presevo municipality (1 ‰) was significantly lower than in Surdulica (13 ‰ and 11 ‰, p <0.05).

In other years of the period there were no statistically significant differences in mortality rates of infants aged 0-6 days were compared between municipalities.

**Figure 2:**
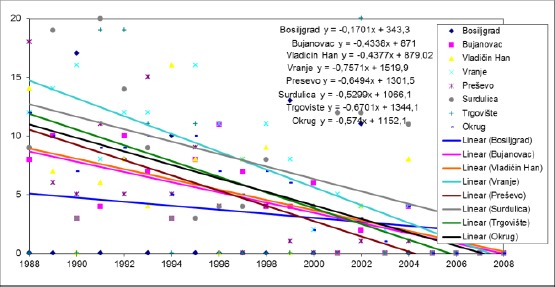
*Trends in the mortality rate of infants aged 0-6 days in the municipalities of Pcinja District from 1988 to 2008*.

In the period from 1988 to 2008, there is a trend of decline mortality rates of infants aged 0-6 days in all municipalities Pčinj districts.

The highest average annual decline in the mortality rate of infants aged 0-6 days from 0.7571 ‰ was recorded in the municipality of Vranje, and this trend was not significantly different from the trend in municipalities Bosiljgrad, where the average annual decline rate was 0.1701 ‰ (p <0, 05) and Bujanovac, where the average annual decline rate was 0.4338 ‰ (p <0.05).

The changes were compared between other municipalities have not happened in significantly different ways.

In 1989, the mortality rate of infants aged 7-28 days in the municipality Bosiljgrad was 25 ‰ and was significantly higher than in Vladicin Han (0 ‰, p <0.05), Vranje (3 ‰, p <0.05) and Presevo (5 ‰, p <0.05).

In 1991, the infant mortality rate 7-28 days of age in the municipality of Vranje (0 ‰) was significantly lower than in the Presevo (6 ‰, p <0.05) and Surdulica (7 ‰, p <0.05).

In other years of the period there were no statistically significant differences in mortality rates of infants aged 7-28 days were compared between municipalities.

**Figure 3:**
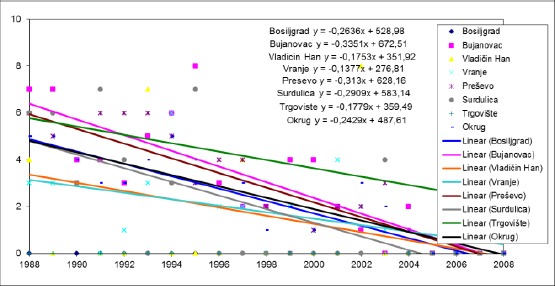
*Trends in the mortality rate of infants aged 7-28 days in the municipalities of Pcinja District from 1988 to 2008*.

In the period from 1988 to 2008, there is a trend of decline mortality rates of infants aged 7-28 days in all municipalities Pčinj districts. The highest average annual decline in the mortality rate of infants aged 7-28 days was recorded in the municipalities of Bujanovac (0.3351 ‰) and Presevo (0.313 ‰) and this decline was significantly higher than that in the municipality of Vranje, which was 0.1377 ‰ (p <0.05). The changes were compared between other municipalities have not happened in significantly different ways. In 1988 the infant mortality rate age 29 days - 2 months in the municipality of Bujanovac was 8 ‰ and was significantly higher than in Vranje (2 ‰, p <0.05). In this year, the highest rate of infant mortality age 29 days - 2 months recorded in the Presevo municipality (11 ‰) and it was significantly higher than in Vranje (p <0.01).

In 1989, the highest mortality rate of infants aged 29 days - 2 months recorded in the municipality of Bujanovac, which was 15 ‰ and was significantly higher than in Vladicin Han (0 ‰, p <0.05), Vranje (2 ‰, p <0.001), Presevo (6 ‰, p <0.05) and Surdulica (0 ‰, p <0.05). In 1991, the highest mortality rate of infants aged 29 days - 2 months was observed in Presevo municipality (7 ‰) and it was significantly higher than in Vranje (1 ‰, p <0.05). In 1998, the highest mortality rate of infants aged 29 days - 2 months recorded in the municipality of Bujanovac, which was 4 ‰ and was significantly higher than in Vranje (0 ‰, p <0.05). In other years of the period there were no statistically significant differences in mortality rates of infants aged 29 days - 2 months were compared between municipalities.

In the period from 1988 to 2008, there is a trend of decline mortality rates of infants aged 29 days - 2 months in all municipalities Pčinj districts.

The highest average annual decline in the mortality rate of infants aged 29 days - 2 months from 0.4766 ‰ was recorded in the municipality of Bujanovac, and this trend was not significantly different from the trend in the municipalities Bosiljgrad, where the average annual decline rate was 0.087 ‰ (p <0 01), Hani Han, where the average annual decline rate was 0.0948 ‰ (p <0.001), Vranje, where the average annual decline rate was 0.1481 ‰ (p <0.001), Pirot, where the average annual decline rate was 0.0883 ‰ (p <0.01) and Targovishte, where the average annual decline rate was 0.1403 ‰ (p <0.05).

**Figure 4:**
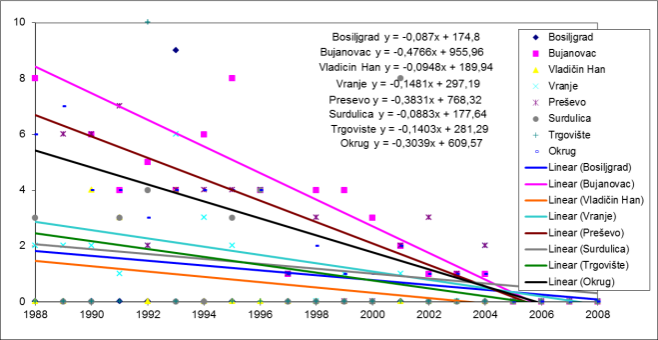
*Trends in the mortality rate of infants aged 29 days - 2 months in the municipalities of Pcinja District from 1988 to 2008*.

The average annual decline in the mortality rate of infants aged 29 days - 2 months in Presevo municipality was 0.3831 ‰, and this trend was not significantly different from the trend in municipalities: Hani Han (p <0.001), Vranje (p <0.01) and Surdulica (p <0.01).

The changes were compared between other municipalities have not happened in significantly different ways.

In 1988 the infant mortality rate aged 2-11 months in the municipality of Bujanovac was 30 ‰ and was significantly higher than in Vladicin Han (0 ‰, p <0.01), Vranje (10 ‰, p <0.001) and Surdulica (9 ‰, p <0.05). In this year, the highest rate of infant mortality aged 2-11 months was recorded in the municipality of Presevo (35 ‰) and it was significantly higher than in Vladicin Han (p <0.001), Vranje (p <0.001), Surdulica (p <0, 05) and Marketplaces (0 ‰, p <0.05).

In 1989, the highest rate of infant mortality aged 2-11 months was observed in Presevo municipality, which was 27 ‰ and was significantly higher than in Vranje (9 ‰, p <0.01) and Surdulica (0 ‰, p <0.01). In the same year mortality rate of infants aged 2-11 months in the municipality of Bujanovac (20 ‰) was significantly higher than in Vranje (p <0.05) and Surdulica (p <0.01).

In 1990, continued the same trend, but the highest rate of infant mortality aged 2-11 months was recorded in the municipality of Presevo (29 ‰) and it was significantly higher than in Vranje (7 ‰, p <0.001) and Surdulica (7 ‰, p <0.05). This year, the mortality rate of infants aged 2-11 months in the municipality of Bujanovac was 24 ‰ and was also significantly higher than in Vranje (p <0.01) and Surdulica (p <0.05).

In 1991, the highest mortality rate of infants aged 2-11 months was recorded in the municipality of Bujanovac (20 ‰) and it was significantly higher than in Vladicin Han (0 ‰, p <0.01), Vranje (4 ‰, p < 0.01) and Surdulica (0 ‰, p <0.01). This year, the mortality rate of infants aged 2-11 months in Presevo municipality was 14 ‰ and it was significantly higher than in Vladicin Han (p <0.05) and Vranje (p <0.05).

And in 1992, the highest rate of infant mortality aged 2-11 months was recorded in the municipality of Bujanovac (27 ‰) and it was significantly higher than in Vladicin Han (4 ‰, p <0.05), Vranje (3 ‰, p <0.001) and Surdulica (7 ‰, p <0.05). This year, the mortality rate of infants aged 2-11 months in Presevo municipality was 20 ‰ and it was significantly higher than in Vranje (p <0.001).

In 1993, the highest mortality rate of infants aged 2-11 months was recorded in the municipality of Presevo (20 ‰) and it was significantly higher than in Vladicin Han (0 ‰, p <0.05), Vranje (8 ‰, p < 0.05) and Surdulica (3 ‰, p <0.05). The mortality rate of infants aged 2-11 months in the municipality of Bujanovac was 16 ‰ and it was significantly higher than in Vladicin Han (0 ‰, p <0.05).

In 1994, the highest mortality rate of infants aged 2-11 months was recorded in the municipality of Bujanovac, where it was 20 ‰ and was significantly higher than in Vladicin Han (0 ‰, p <0.05), Vranje (1 ‰, p <0.001) and Surdulica (0 ‰, p <0.01). In the same year mortality rate of infants aged 2-11 months in Presevo municipality was 15 ‰ and was significantly higher than in Vranje (p <0.001) and Surdulica (p <0.05).

And in 1995, the highest rate of infant mortality aged 2-11 months was recorded in the municipality of Bujanovac, which was 17 ‰ and was significantly higher than in Vladicin Han (0 ‰, p <0.05) and Vranje (1 ‰, p <0.001). In the same year mortality rate of infants aged 2-11 months in Presevo municipality was 12 ‰ and was significantly higher than in Vranje (p <0.001).

And in 1996, the highest rate of infant mortality aged 2-11 months was recorded in the municipality of Bujanovac, which was 17 ‰ and was significantly higher than in Vladicin Han (0 ‰, p <0.05) and Vranje (2 ‰, p <0.001). In the same year mortality rate of infants aged 2-11 months in Presevo municipality was 11 ‰ and was significantly higher than in Vranje (p <0.05).

In 1998, the mortality rate of infants aged 2-11 months in the municipality of Bujanovac was again significantly higher than in Vranje (10: 2 ‰, p <0.05).

In 1999, the infant mortality rate aged 2-11 months is the highest in the municipality Bosiljgrad (26 ‰) and was significantly higher than in Vranje (2 ‰, p <0.05).

In 2001, the infant mortality rate aged 2-11 months in the municipality of Vranje was 1 ‰, this value is significantly lower than in Bujanovac (7 ‰, p <0.05), Vladicin Han (14 ‰, p <0.05) and Presevo (9 ‰, p <0.05).

In 2007, the highest mortality rate of infants aged 2-11 months was recorded in the municipality of Vladičin Han, which amounted to 31 ‰ and was significantly higher than in Bujanovac (4 ‰, p <0.05), Vranje (5 ‰, p <0.01) and Presevo (5 ‰, p <0.01).

In other years of the period there were no statistically significant differences in mortality rates of infants aged 2-11 months were compared between municipalities.

**Figure 5:**
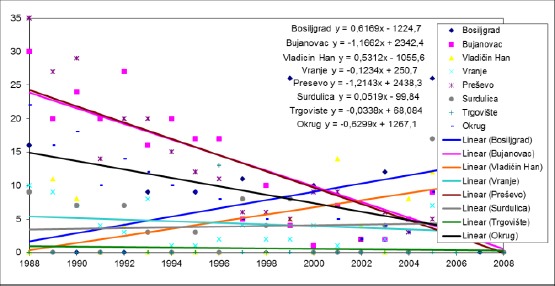
*Trends in the mortality rate of infants aged 2-11 months in the municipalities of Pcinja District from 1988 to 2008*.

In the period from 1988 to 2008, there is a trend of decline mortality rates of infants aged 2-11 months in Presevo (average annual decline in the mortality rate of 1.2143 ‰), Bujanovac (a decrease of 1.1662 ‰), Vranje (decline from 0.1234 ‰) and market (down from 0.0338 ‰). The rising trend in mortality rates of infants aged 2-11 months was recorded in the municipalities Bosiljgrad (average annual increase in the mortality rate of 0.6169 ‰), Hani Han (an increase of 0.5312 ‰) and Surdulica (an increase of 0.0519 ‰). The trends recorded in Presevo and Bujanovac were statistically significantly different from the trends in the municipalities Bosiljgrad (p <0.001), Hani Han (p <0.001), Vranje (p <0.001), Pirot (p <0.001) and Targovishte (p < 0.001). The change in Vranje was significantly different from the trend in the municipalities Bosiljgrad (p <0.05) and Hani Han (p <0.05). The change in Targovishte was significantly different from the trend in Vladicin Han (p <0.05). The changes were compared between other municipalities have not happened in significantly different ways.

In conclusion, toxic metals, having reached to the organism, with their physico-chemical properties, cause temporary or permanent damage to the structure or function of one or more organs or systems. Into the organism can be entered, both in increased concentrations currently or at shorter intervals, and in low concentrations over a longer period of time. The effect on the organism depends on their nature, time exposure and individual characteristics of the organism clinical aspect of toxic metal poisoning may be acute, subacute and chronic.

In professional terms acute poisoning is rare and occurs in accident situations, in case of entered greater amounts of toxic metals in brief period of time period. Chronic occupational poisonings are more common and their clinical picture depends upon the quantity of entered toxic metals, entering paths and individual features of the exposed individuals, %, gender, age, physiological conditions, the existence of intercurrent diseases, previous sick conditions, genetic variation and others. However, acute clinical manifestations may occur after prolonged exposure, such as the clinical and chronic effects may occur after short-term acute exposure.

Ecotoxicology with toxicology studies direct or indirect effects of xenobiotics on the ecosystem, in all living organisms and their organization, attitude toward inanimate matter, interrelationships and attitude towards the man. Nutritional supplements of Vitamin E and selenium in combination can reduce the toxicity of certain heavy metals, such as: mercury, which is found in fish, according to a new study conducted in our laboratory in the country. Accumulation of heavy metals in components of agroecosystems is caused by the intensity of precipitation, soil characteristics, mineral composition of the substrate and type of the grown crops. Soil and groundwater in agricultural ecosystems play an important role in the retention of heavy metals, primarily due to their role as filters and buffers. Mechanisms of chemical immobilization in the surface layer of the soil defines the retention of heavy metals, and further transport through the soil profile depends on the geochemical and soil processes related to a specific vegetation and habitats edaphic conditions.

By using appropriate methodological principle it is possible to predict the occurrence and critical levels of hazardous pollutants in order to repair their negative effects on the environment. Using laboratory methods, statistical information systems and selection of appropriate fertilizers and agricultural technology to a large extent can mitigate the toxic effects of heavy metals in the agroecosystem [24-26].
